# Selecting contextually appropriate performance indicators in a circumpolar context: a modified Delphi approach

**DOI:** 10.1186/s12913-021-06485-2

**Published:** 2021-05-30

**Authors:** Rebecca Rich, Thomsen D’Hont, Kellie E. Murphy, Jeremy Veillard, Susan Chatwood

**Affiliations:** 1 Department of Obstetrics & Gynaecology, University of Alberta, Royal Alexandra Hospital, 5S Robbins Pavilion, 10240 Kingsway Avenue, Edmonton, AB T5H 3V9 Canada; 2 Department of Family Medicine, University of Alberta, Suite 205 College Plaza 8215 - 112 St, Edmonton, AB T6G 2C8 Canada; 3 Division of Maternal Fetal Medicine, Obstetrics & Gynaecology, University of Toronto, Mount Sinai Hospital, Room 3-918, 700 University Avenue, Toronto, ON M5G 1Z5 Canada; 4 Institute of Health Policy, Management and Evaluation, Dalla Lana School of Public Health, University of Toronto, 155 College Street, Suite 425, Toronto, ON M5T 3M6 Canada; 5 School of Public Health, University of Alberta, 3-279 Edmonton Clinic Health Academy, 11405 - 87 Ave NW, Edmonton, AB T6G 1C9 Canada

**Keywords:** maternal health, infant health, Arctic regions, Indigenous health services, health care quality indicators

## Abstract

**Background:**

Meaningful performance measurement requires indicators to be scientifically robust and strategically focused. For many circumpolar states, indicators aligned with national strategies may ignore the priorities of northern, remote, or Indigenous populations. The aim of this project was to identify contextually appropriate performance indicators for maternity care in circumpolar regions.

**Methods:**

Fourteen maternity care and health systems experts participated in a modified Delphi consensus process. The list of proposed indicators was derived from a previously published scoping review. Fourteen participants rated each proposed indicator according to importance, circumpolar relevance, validity, and reliability and suggested additional indicators for consideration.

**Results:**

Consensus was achieved after two rounds, as measured by a Cronbach’s alpha of 0.87. Eleven indicators, many of which represented physical health outcomes, were ranked highly on all four criteria. Twenty-nine additional indicators, largely focused on social determinants of health, health care responsiveness, and accessibility, were identified for further research. Travel for care, cultural safety and upstream structural determinants of health were identified as important themes.

**Conclusions:**

This study identified the important gaps between current performance measurement strategies and the context and values that permeate maternal-child health in circumpolar regions. The indicators identified in this study provide an important foundation for ongoing work. We recommend that future work encompass an appreciation for the intersectoral nature of social, structural, and colonial determinants of maternal-child health in circumpolar regions.

**Supplementary Information:**

The online version contains supplementary material available at 10.1186/s12913-021-06485-2.

## Background

Among health systems around the world, performance measurement has become an increasingly popular tool in the pursuit of health care quality, accountability, and value for money [1–4]. This trend has been accompanied by an explosion in the number of available performance indicators and a dramatic increase in the number of organizations collecting and reporting on health system performance information. In this environment, meaningful performance measurement requires indicators that are scientifically robust and strategically focused. Indicators must be valid, reliable, sensitive to change, and feasible to measure. They must also be aligned with the values and priorities of the system they are intended to evaluate [5–7].

For circumpolar states, those whose political boarders encompass the most northern regions of the globe, the selection of contextually relevant indicators presents unique challenges. Geopolitically, “circumpolar” refers to all of Alaska, Greenland, Iceland, the Faroe Islands, and the northernmost territories of Sweden, Finland, Norway, Russia and Canada. Of course, these regions are diverse in their geography, populations and health systems, and some areas experience more health inequities than others. Despite their differences, they share many challenges such as vast distances, harsh winter climates, and low population densities, all of which make the delivery of health care challenging and expensive. Circumpolar health systems thus rely more heavily on long distance travel of both patients and providers than do their southern counterparts. In addition, many circumpolar regions have large Indigenous populations which comprise up to 90 % of the population in some regions. It is thus imperative to consider the historical and ongoing impacts of settler colonialism and the substantial health inequities that exist both within and between circumpolar regions and disproportionately affect Indigenous peoples and communities.

Maternity care provides a useful example of the unique health care landscape in circumpolar regions. While medical evacuation can provide critical access to secondary and tertiary level care and is an important component of all rural and remote health systems, efforts at centralization have also put the sustainability of local birth programs at risk. The resultant practice of routinely evacuating low-risk women for labour and birth has substantial and well-documented consequences for patients, families and communities [8–11]. Where possible, it is considered best practice to provide care for labour and birth closer to home [12, 13]. Being a uniquely rural/remote phenomenon, this issue affects a minority of women in Canada and other circumpolar states and is not often captured in pan-national performance measurement efforts [14, 15]. Canadian maternity care indicators, for example, focus largely on decreasing practice variation with respect to obstetrical interventions, such as induction of labour, operative vaginal delivery and caesarean section. This approach has positioned cost savings and improvements in patient safety through a reduction in intervention as central performance targets [16]. Pan-national indicators such as these are often selected based on the strategic priorities of a dominant southern majority and may ignore or even conflict with the needs and priorities of northern or remote communities. This is particularly relevant for health systems serving Indigenous communities as the importance of including Indigenous knowledge and stakeholder input in the selection of performance indicators has been well documented in the literature [17, 18]. This study attempts to begin filling this gap by identifying performance indicators which are contextually appropriate for circumpolar health systems.

## Methods

 A two-round modified Delphi consensus study was conducted among circumpolar maternity care experts. The objectives of the study were to establish consensus on indicator priorities and identify contextually relevant perinatal performance indicators. The Delphi method is frequently applied in circumstances where the available evidence is contradictory or insufficient [19] and is a common method for the selection of key performance indicators [1, 20–23].

This study was carried out in accordance with the Tri-Council Policy Statement: Ethical Conduct for Research Involving Humans. Particular attention was devoted to Chap. 9, Research Involving the First Nations, Inuit and Métis Peoples of Canada [24]. There was consultation throughout the project with a First Nations Elder, medicine woman and healer who has extensive experience working with health researchers. This study was approved by the University of Toronto research ethics board. Territorial research licenses were obtained from the Aurora and Nunavut Research Institutes.

### Participant selection

Participants were selected using purposeful sampling as the quality of results produced by a Delphi panel depends on the expertise of participants [25]. First, an advisory committee was assembled which included experts with extensive circumpolar networks. Each member recommended colleagues for participation. Invited participants were required to be experts in the field of maternal-child health either through clinical work, research, health policy, and/or Indigenous Traditional Knowledge. We sought out a heterogeneous panel to ensure that a wide variety of stakeholder groups were represented. A target sample size of 10–20 was chosen to balance the trade-offs between decision quality and data manageability as well as between panel heterogeneity and the ability to achieve consensus.

Many, but not all, circumpolar regions and types of expertise were represented. The group included two representatives from Alaska, one from the Yukon Territory, two from the Northwest Territories, four from Nunavut, two from Greenland, and three who currently work in southern Canada but have significant expertise in Arctic or circumpolar maternal-child health. The group included physicians, midwives, nurses, and public health researchers. Many of the participating clinicians currently work in research or health policy roles, allowing them to bring multiple areas of expertise to the panel.

### Derivation of survey items

The questionnaires used were developed for this consensus process. Survey items were derived from a scoping review, which has been published elsewhere [15]. Sixty-two unique maternity care performance indicators were identified through this review and subsequently subdivided into the following domains according to a modified version of the OECD Health Performance Framework [26]: Determinants of health, health outcomes, health system effectiveness, safety, responsiveness, accessibility, and cost. It should be noted that the terminology used for each indicator title was maintained from the original source and does not reflect the preferred langugage of the authors.

### Response scale and criteria

Respondents were asked to rate each of the 62 indicators on a 7-point Likert-type scale ranging from 1 (strongly disagree) to 7 (strongly agree) according to the following four criteria:

- Importance: The level of concern of health care users or policy makers and the degree to which the indicator is susceptible to influence by the health system.
- Circumpolar relevance: The significance of the indicator within the participant’s circumpolar context.
- Validity: The degree to which the indicator measures what it intends to measure.
- Reliability: The degree to which the indicator provides stable results across various populations, circumstances, and time points.

In order to allow participants to best assess the validity and reliability of each indicator, a detailed information package was provided to each participant. During the first round, participants were also asked to suggest additional indicators not identified in the literature review. These additional indicators were incorporated into the second-round survey for evaluation by the panel^2^.

### Survey administration

The questionnaires were created and distributed electronically. Two researchers piloted the questionnaire to ensure it was clearly worded and the suggested time for completion was appropriate. Responses to the first-round survey were collected over a period of 41 days. Responses were collected and tabulated to create the second-round survey, which included the same 62 indicators along with the 17 additional indicators suggested by participants. Round-two responses were collected over a period of 19 days.

### Data analysis

After collection of the first-round responses, measures of central tendency and dispersion (mean, median and standard deviation (SD)) were calculated for each indicator against each criterion. This information was included as structured feedback in the second round of the survey. Consensus among participants was determined by the degree of internal consistency demonstrated by participant responses. This was established using standardized Cronbach’s alpha. This method assumes that each indicator possesses a true level of each characteristic. Each participant’s response to a given survey question represents a single measurement of that indicator’s importance, relevance, validity, or reliability. The internal consistency of responses can thus be considered a measure of agreement among participants. Published thresholds for an appropriate Cronbach’s alpha vary in the literature from 0.70 [27] for the determining the inter-rater reliability of a psychometric scale designed to differentiate between groups to 0.90 [28] for use of a diagnostic scale on an individual level. The threshold for determining consensus among experts on a Delphi panel is similarly variable but a cut-off of α ≥ 0.8 is frequently used [29, 30]. All statistical calculations were performed using SAS University Edition.

### Selection of key indicators

After achievement of consensus, indicators for which ≥80 % of participants selected 6 (agree) or 7 (strongly agree) on all four criteria were identified as a core set of indicators. Using importance and circumpolar relevance as gateway criteria, a second set of indicators was also identified for consideration. The latter group included indicators for which ≥80 % of participants provided a strong rating (6 or 7) for the criteria of importance and relevance but that did not meet the same threshold for validity and reliability.

## Results

### Achieving consensus

Twenty-one experts were invited to participate in the Delphi consensus process. Fourteen experts (67 %) completed two rounds of the Delphi process. The level of agreement among participants, as measured by standardized Cronbach’s alpha, is displayed in Table 1. After two rounds, the level of agreement was very good as demonstrated by a Cronbach’s alpha of 0.87. Missing values accounted for 1.3 % of all response options.

**Table 1:** Level of Agreement Among Delphi Participants

Round	Participants	Agreement (Cronbach’s α)		
		Missing values excluded	Missing values replaced with neutral value (4)	Missing values replaced with mean value
**Round 1**	14	0.79	0.75	0.79
**Round 2**	14	0.87	0.86	0.87

Standardized Cronbach’s α calculated based on management of missing data. Consensus achieved when α $\ge$0.8

### Selected indicators

Once consensus had been reached, the proportion of responses indicating participant agreement (6 - agree or 7 - strongly agree) was tabulated for each survey statement. Eleven indicators were rated highly on all four criteria by ≥ 80 % of the participants and have been labeled the “core” indicators (Table 2). This set of indicators primarily focuses on physical health outcomes but does contain four indicators which represent other domains of health system performance. These additional four indicators are as follows: teenage pregnancy and birth, admissions to the neonatal intensive care unit (NICU), presence of a skilled birth attendant, and travel to place of birth.

**Table 2:** Selected “Core” Performance Indicators

Indicator	Ratings ≥ 6 (%)			
	**Importance**	**Relevance**	**Validity**	**Reliability**
**Determinants of health**				
Teenage pregnancy & birth	93	93	93	93
**Health outcomes**				
Anemia in pregnancy	93	93	86	86
Stillbirths	100	93	93	93
Perinatal deaths	100	100	100	100
Preterm birth	100	100	86	86
Maternal Mortality	100	93	100	100
Neonatal mortality	100	100	100	100
Low birth weight	86	93	93	93
**Effectiveness**				
NICU admissions	100	85	100	100
**Accessibility**				
Birth attendant	93	93	93	93
Travel to place of birth	92	93	86	86

Indicators for which > 80 % of participants provided a rating of 6 or 7 for each criterion

Twenty-nine additional indicators received high ratings for importance and relevance but lower ratings for reliability or validity, indicating that further study or development of these indicators may be required (Table 3). It is important to note that this group of indicators includes a much greater focus on social determinants of health, health system responsiveness and health care accessibility. Three major themes are represented within this second group of indications: Increased focus on the social determinants of health; the impact of medical travel; and the importance of culturally appropriate care at both provider and system levels.

**Table 3:** Selected “Additional” Performance Indicators

Indicator	Ratings ≥ 6 (%)			
	**Importance**	**Relevance**	**Validity**	**Reliability**
**Determinants of health**				
Maternal education level	93	93	93	79
Domestic violence	93	100	21	7
Smoking during pregnancy	100	100	57	50
Use of illicit drugs during pregnancy	100	93	21	14
Use of alcohol during pregnancy	100	100	29	21
Breastfeeding practices	100	100	64	43
Food insecurity^a^	100	100	-	-
Maternal housing (crowded or under-housed)^a^	93	93	-	-
**Health outcomes**				
Diabetes in pregnancy	93	93	86	79
Postpartum hemorrhage	93	93	71	50
Postpartum depression	86	93	29	21
Congenital anomalies	86	86	86	79
Small for GA infants	100	100	86	79
**Effectiveness**				
Neonatal readmission to hospital	86	86	93	79
**Safety**				
Births without obstetric intervention	93	86	64	64
Transfers for obstetrical indications^a^	92	85	-	-
Unplanned births in the community^a^	100	92	-	-
**Responsiveness**				
Characteristics of care providers	92	93	69	69
Cultural competency	92	86	46	38
Patient reported unfair treatment based on ethnicity	92	86	57	29
Patient reported support during labour and birth	100	86	50	50
Presence of breastfeeding support programs	92	86	29	14
Patient reported satisfaction with care	100	100	50	36
**Accessibility**				
Frequency and timing of antenatal care	93	93	79	71
Use of antenatal ultrasound	86	86	93	79
Rate of induced abortions	86	86	86	79
Postpartum visits	100	86	71	64
Maternity care provider in patient’s community^a^	100	93	-	-
Maternity care provider who speaks the same language/is from the same culture as the patient^a^	92	93	-	-

Indicators for which ≥ 80 % of participants provided a rating of 6 or 7 on the basis of importance and circumpolar relevance

^a^Denotes additional indicator suggested by members of the Delphi panel in the first round

## Discussion

### Selected indicators

This study demonstrates the ability of a modified Delphi approach to achieve consensus among a geographically dispersed and professionally diverse group of stakeholders from circumpolar regions. The eleven selected core indicators (Table 2), are largely focused on morbidity and mortality. This is consistent with the findings of our earlier work [15] and reflects the historical dominance of physical health outcomes in performance measurement and health surveillance systems. Physical health outcomes provide the most straightforward opportunity for standardizing definitions, collecting high quality data, and supporting interregional comparisons. The validity and reliability of these indicators is also well established in the literature. However, they capture only a tiny fraction of health system performance. Frameworks, such as the OECD framework utilized in this study, exist in order to capture other domains of health system performance and ensure performance measurement strategies are adequately thorough. It is clear from the narrow selection of core indicators identified in this study, that a complete set of scientifically robust, strategically focused, and contextually appropriate performance indicators does not yet exist for circumpolar maternity care systems.

Consistent with this conclusion, is the identification of 29 additional indicators for further consideration (Table 3). These indicators, which were identified using importance and relevance as gateway criteria, but did not require evidence of strong scientific validity or reliability, demonstrate the importance of evaluating circumpolar health systems through the domains of social determinants of health, healthcare accessibility, and health system responsiveness. The decision to include an entire second set of indicators based on these gateway criteria rests on their significant importance in concert with the absence of available scientific evidence demonstrating validity and reliability. They therefore provide critical insight into circumpolar priorities while highlighting some of the challenges associated with circumpolar health system performance measurement. The complex nature of some of the suggested constructs, particularly those necessitating patient reported outcomes, introduces the need for rigorous development of these indicators which has yet to be documented in the literature. Additional barriers include a lack of availability of data sources to support these indicators and the reticence of health system leadership to direct resources toward development of indicators for small populations. The critical, value-based themes that weave throughout this list reflect the predominance of travel for care within circumpolar health systems and the limited availability of culturally appropriate care for Indigenous patients and families in many regions.

Social and structural determinants of health such as income, education, social supports, gender, and experiences of racism and colonization are all known to influence health behaviours, health system access and health outcomes [31–34]. In addition, social and structural determinants of health contribute to disadvatage and health inequity in an intersectional fashion. Examples of such interconnected and overlapping determinants of maternal-child health are highlighted in this study and include maternal substance use, maternal education, the presence or absence of intimate partner violence, food insecurity and insecure housing. While there is strong evidence that communities in some circumpolar regions are disproportionately affected by these challenges, they are not routinely included in perinatal health surveillance systems. This is a direct reflection of the fact that health system performance and healthcare quality initiatives are typically siloed from the social and political systems which impact these upstream determinants.

In many circumpolar regions, structural, political, and social determinants of health are further compounded by physical isolation, harsh winter climates, increased industrialization, and changing environmental conditions. These factors contribute to the geographical and logistical barriers that patients face when accessing healthcare. Multiple indicators of healthcare access were highlighted in this study. These include but are not limited to the number of antepartum or postpartum visits a patient is able to attend, the availability of obstetrical ultrasound, and the distance a patient is required to travel for care. The latter indicator was identified from the Canadian Maternity Experiences Survey [35] and captures the proportion of women who report traveling > 100 km to receive care. It is important to consider the fact that in many circumpolar regions around the world, distances are almost always greater than 100 km. With this in mind, the Delphi panel suggested other measures of travel for care including the proportion of women who can access a maternity care provider (and/or skilled birth attendant) in their community, the number of unplanned births occurring in each community (which generally occur as a result of patients being unable or unwilling to travel for birth), and the number of emergency transfers for obstetrical indications. If “distance” to care is measured within a circumpolar context, we suggest using the number of “days or hours away from home” as a more appropriate reflection of the travel burden. The Delphi panel also suggested that an indicator be developed to capture the proportion of women in a region receiving care in their own language. This, of course, reflects the fact that accessibility is not equivalent to geographical proximity and equitable access to care requires that systems and providers minimize social, financial and systematic barriers to care and ensure the availability of culturally safe care for Indigenous women.

Health system responsiveness is a concept intended to capture the ability of a health system to acknowledge, accommodate, and react to the expectations of the population. It shares many tenants with patient centered care and overlaps with the principle of cultural safety [36]. For Indigenous women, providing culturally appropriate and thus responsive care may necessitate a holistic appreciation of health [37, 38] including physical, mental, emotional, spiritual, and historical considerations. It is well established that racism, colonization, and presence or absence of access to self-determination are important determinants of Indigenous peoples’ health [10, 39, 40]. The Delphi panel demonstrated this critical issue by selecting multiple indicators reflective of cultural competency or safety or other aspects of the patient experience. Their suggestions included a number of patient reported outcomes reflecting the necessity to assess cultural safety through the eyes of the individual, family or community receiving care. This study suggests that health care providers, institutions and health system leaders should not only prioritize efforts to provide culturally safe care, but should also measure and report on their ability to do so. A further critique of how the study findings relate to Indigenous peoples’ health and to cultural safety is included below.

### Data sources and feasibility

Beyond the specific indicators identified by the Delphi panel, our findings highlight some important challenges and opportunities for circumpolar performance measurement initiatives. First, when reporting rare outcomes among small populations, aggregation of data over large regions or timeframes may be required to ensure confidentiality and statistical rigor. Thus, collection and reporting of such indicators within the circumpolar context may not always be feasible and there may be a larger role for process indicators and confidential audits of rare outcomes such as maternal and neonatal mortality. Second, health system performance measurement is only as effective as the data that supports it. In the Canadian territories, for example, there is limited prospective collection of perinatal surveillance data. There is also a marked lack of Indigenous specific identifiers within Canadian health data sources more broadly [41, 42]. The development and implementation of Indigenous health information systems in Canada is essential and must be carried out by and with Indigenous communities and organizations to ensure appropriate indicator selection, data usage and governance [43, 44]. Finally, and most importantly, our findings reinforce the fundamental role of context and values within health system stewardship [6, 17, 45] and the importance of aligning health policy with broader agendas [46]. In Canada, we have seen the recent adoption of the UN Declaration on the Rights of Indigenous Peoples and the release of the final reports of the Truth and Reconciliation Commission and the Inquiry into Missing and Murdered Indigenous Women and Girls [47–50]. In association with these reports, Canadian National bodies have begun to recognize the importance of Indigenous rights and self-governance. Despite this, the commonly used performance measurement systems are still incongruent with these values. Canadian policy makers thus have an opportunity and an obligation to engage with northern stakeholders and Indigenous communities to ensure that health policies and resources are directed appropriately. Furthermore, when developing performance measurement strategies intended to capture and address health inequities, it is critical to recognize the role that defect-based approaches to health surveillance have had and continue to have on the stigmatization of Indigenous peoples within the health literature. Adequate engagement and attention to strengths-based approaches can limit this unintended consequence while simultaneously providing opportunities for health system improvement and management.

### Limitations

The authors recognize a number of limitations which we hope will generate important reflection and discussion for future efforts among all who work in this field. While many clinicians, researchers and health policy makers in circumpolar regions communicate regularly in English, the exclusion of other languages from this study limited the potential pool of Nordic, Russian, Greenlandic, and North American Indigenous participants. Furthermore, because performance measurement efforts are typically centralized in southern centers, and are dominated by non-Indigenous individuals, capacity and expertise among circumpolar and Indigenous experts is limited to a small number of exceedingly busy people. The composition of the Delphi panel was thus heavily weighted toward the North American settler experience, and readers should be aware of the impact this may have on generalizability of the findings.

A discussion of health system performance in circumpolar regions would not be complete without an acknowledgement of the coloniality of health care and of health system performance measurement strategies more broadly. While the project’s foundation was heavily influenced by post-colonial and critical race theories, health equity, and *Etuaptmumk* (two-eyed seeing), the authors humbly recognize that this study uses entirely western methodologies and a western health system performance framework. Based on this experience, we offer our reflections on the ideological and practical implications of this approach.

We have previously named experiences of colonization and anti-Indigenous racism as important determinants of Indigenous peoples’ health. While this applies directly to the importance of selecting indicators of cultural safety at the level of the patient-provider interaction and within health system structures, it should also be recognized that health system surveillance and health system performance measurement efforts, including the methodologies used in this project, are products of a colonial system. A more ethical, effective, and generalizable approach would have been to, under the direction of the Elders involved in this project, apply Indigenous methodologies and patient/community level engagement alongside or in place of the Delphi consensus process.

### Recommendations and future directions

While many of the selected indicators are not ready for implementation as they have been presented here, circumpolar health regions interested in improving their use of contextually relevant performance indicators, may find this a useful resource.

In order to prepare these indicators for use we recommend that further indicator development be carried out in consultation with patients, families, community leaders and other local stakeholders. Concomitant development and application of region-specific or circumpolar health system performance frameworks will help to maintain comprehensiveness and relevance while minimizing indicator redundancy. Where the health and wellness of Indigenous families or communities is relevant, we recommend performance measurement strategies be developed by and with Indigenous communities and leaders and utilize locally appropriate methodologies for information gathering and interpretation.

While we believe the findings of this study are useful at face value, we hope that the above discussion of the broader colonial context within which it was conducted will help to generate important consideration, reflection, and a collective effort to advance contextually and culturally appropriate, value-based, responsive, and community driven work within circumpolar health system performance measurement.

## Conclusions

In this study, we identified eleven scientifically robust maternity care indicators that are important and relevant within the circumpolar context. We also identified many indicators that more broadly reflect the priorities in circumpolar regions and warrant further consideration and development. This finding is demonstrative of the significant gap that currently exists between performance measurement strategies and the context and values in circumpolar regions.

Based on the findings of this study, we recommend that circumpolar health system leaders re-evaluate their current perinatal performance measurement systems to ensure responsiveness and contextual appropriateness. The indicators identified in this study may provide an important foundation for this work, but should not be implemented without robust community and stakeholder engagement at local and regional levels as well as the development and application of circumpolar or region-specific health system performance frameworks.

While we recognize that successful health system performance measurement strategies require centralization and oversight, we recommend that future efforts encompass an appreciation for the intersectoral nature of social, structural, and colonial determinants of maternal-child health in circumpolar regions. The necessity for cooperation between the health and social services sectors and the value of local and Indigenous knowledge cannot be overstated.

It is our hope that ongoing work in this area will ultimately lead to the development of intersectional, value-based, and contextually appropriate maternal-child health indicators which are supported by data systems with appropriate ownership and governance. Only with this approach can we foster continuous quality improvement in circumpolar maternity centers, offer opportunities for inter-regional comparisons, and support evidence-based drivers towards health equity.

## Supplementary Information

**Additional file 1. **Delphi Questionnaire Round 2. As outlined in the methods section, and is standard in a Delphi consensus process, the survey instruments used were designed for the purpose of this study. Survey item derivation is described above. This attached additional file includes a copy of the second round questionnaire as well as the associated preamble as it was distributed to Delphi panel members. The second round questionnaire has been included alone as it encompasses the both the contents of the first round questionnaire as well as the summary statistics generated in the first round and the additional indicators suggested by the Delphi panel in round 1. It thus provides a complete representation of the survey items distributed to panel members.

## Data Availability

The datasets used and/or analysed during the current study are available from the corresponding author on reasonable request.

## Abbreviations

OECD: Organization for Economic Cooperation and Development
NICU: Neonatal intensive care unit
GA: Gestational age
NWT (or NT): Northwest Territories
NU: Nunavut

## Footnotes

2 The second round survey has been included as a supplementary file.

## References

[1] Carinci F, Van Gool K, Mainz J, Veillard J, Pichora E, Januel J (2015). Towards actionable international comparisons of health system performance: expert revision of the OECD framework and quality indicators. Int J Qual Health Care.

[2] Freeman T (2002). Using performance indicators to improve health care quality in the public sector: a review of the literature. Health Serv Manag Res.

[3] Murray C, Evans D (2003). Health systems performance assessment: debates, methods and empiricism.

[4] Smith PC, Mossialos E, Papanicolas I. Performance measurement for health system improvement: experiences, challenges and prospects. 2008.

[5] Brown AD, Veillard J, Commentary (2008). Indicators with a Purpose–Meaningful Performance Measurement and the Importance of Strategy. Healthcare Policy.

[6] Veillard J, Huynh T, Ardal S, Kadandale S, Klazinga NS, Brown AD (2010). Making health system performance measurement useful to policy makers: aligning strategies, measurement and local health system accountability in Ontario. Healthcare Pol.

[7] World Health Organization. The world health report 2000: health systems: improving performance. World Health Organization; 2000.

[8] Chamberlain M, Barclay K (2000). Psychosocial costs of transferring indigenous women from their community for birth. Midwifery.

[9] Lawford K, Giles AR (2012). An analysis of the evacuation policy for pregnant First Nations women in Canada. AlterNative.

[10] Moffitt PM, Colonialization (2004). A health determinant for pregnant Dogrib women. J Transcult Nurs.

[11] Varcoe C, Brown H, Calam B, Harvey T, Tallio M. Help bring back the celebration of life: A community-based participatory study of rural Aboriginal women’s maternity experiences and outcomes. BMC Pregnancy and Childbirth. 2013;13. 10.1186/1471-2393-13-26 PMC3577503 23360168

[12] Lalonde A, Grzybowski SC, Klein MC, Iglesias S, Gagne GP (1998). Rural obstetrics: joint position paper on rural maternity care. Can J Rural Medicine.

[13] Miller KJ, Couchie C, Ehman W, Graves L, Grzybowski S, Medves J (2012). Rural maternity care. J Obstetr Gynaecology Canada.

[14] Canadian Institute for Health Information (CIHI) (2013). Canadian Institute for Health Information. Health Indicators 2013.

[15] Rich R, D’Hont T, Linton J, Murphy KE, Veillard J, Chatwood S. Performance Indicators for Maternity Care in a Circumpolar Context: A Scoping Review. Int J Circumpolar Health. 2016;75 (31470). 10.3402/ijch.v75.31470 PMC5149666 27938636

[16] Canadian Institute for Health Information (CIHI). Canadian Insititute for Health Information: Indicator Library 2015 [cited 2015 Nov]. Available from: http://indicatorlibrary.cihi.ca/display/HSPIL/Indicator+Library?desktop=true.

[17] Chatwood S, Bytautas J, Darychuk A, Bjerregaard P, Brown A, Cole D, et al. Approaching a collaborative research agenda for health systems performance in circumpolar regions. Int J Circumpolar Health. 2013;72. 10.3402/ijch.v72i0.21474 PMC3745603 23961514

[18] Anderson M, Smylie J, Anderson I, Sinclair R, Crengle S. First Nations, Metis, and Inuit Health Indicators in Canada: A background paper for the project “Action Oriented Indicators of Health and Health Systems Development for Indigenous Peoples in Australia, Canada, and New Zealand”. The Indigenous Peoples’ Health Research Centre (IPHRC); 2006.

[19] Graham B (2003). Delphi as a method to establish consensus for diagnostic criteria. J Clin Epi.

[20] Zeitlin J, Wildman K, Breart G, Alexander S, Barros H, Blondel B (2003). PERISTAT: indicators for monitoring and evaluating perinatal health in Europe. Eur J Public Health.

[21] Boulkedid R, Sibony O, Goffinet F, Fauconnier A, Branger B, Alberti C (2013). Quality Indicators for Continuous Monitoring to Improve Maternal and Infant Health in Maternity Departments: A Modified Delphi Survey of an International Multidisciplinary Panel. PLoS ONE.

[22] Gagliardi AR, Fung MFK, Langer B, Stern H, Brown AD (2005). Development of ovarian cancer surgery quality indicators using a modified Delphi approach. Gynecol Oncol.

[23] Boulkedid R, Abdoul H, Loustau M, Sibony O, Alberti C (2011). Using and reporting the Delphi method for selecting healthcare quality indicators: a systematic review. PLoS ONE.

[24] Canadian Institutes of Health Research, Natural Sciences and Engineering Research Council of Canada. Social Sciences and humanities Research Council of Canada. Tri-Council Policy Statement: Ethical Conduct for Research Involving Humans. Ottawa, Canada; 2014.

[25] Hsu C-C, Sandford BA. The Delphi Technique: Making Sense Of Consensus. Practical Assessment, Research & Evaluation. 2007;12(10).

[26] Arah OA, Klazinga N, Delnoij D, Ten Asbroek A, Custers T (2003). Conceptual frameworks for health systems performance: a quest for effectiveness, quality, and improvement. Int J Qual Health Care.

[27] Nunnally JC, Bernstein I (1994). The assessment of reliability. Psychometric Theory.

[28] Bland JM, Altman DG (1997). Statistics notes: Cronbach’s alpha. BMJ.

[29] Zevin B (2014). Design and Validation of a Comprehensive Simulation-Enhanced Training Curriculum for a Complex Minimally.

[30] Shore E (2014). Design of a Comprehensive Evidence-Based Laparoscopy Curriculum for Gynecoogy Residents.

[31] Canadian Institute for Health Information (CIHI) (2013). A performance measurement framework for the Canadian health system.

[32] Marmot M, Friel S, Bell R, Houweling TA, Taylor S, Commission on Social Determinants of Health (2008). Closing the gap in a generation: health equity through action on the social determinants of health. Lancet.

[33] Dahlgren G, Whitehead M (1991). Policies and strategies to promote social equity in health.

[34] Paradies Y, Ben J, Denson N, Elias A, Priest N, Pieterse A (2015). Racism as a determinant of health: a systematic review and meta-analysis. PloS one.

[35] Public Health Agency of Canada. What Mothers Say: The Canadian Maternity Experiences Survey. Ottawa; 2009.

[36] Saha S, Beach MC, Cooper LA (2008). Patient centeredness, cultural competence and healthcare quality. J Natl Med Assoc.

[37] Smylie J, Anderson I, Ratima M, Anderson M (2006). Indigenous health performance measurement systems in Canada, Australia, and New Zealand. The Lancet.

[38] Stephens C, Nettleton C, Porter J, Willis R, Clark S (2005). Indigenous peoples’ health—why are they behind everyone. everywhere? The Lancet.

[39] Allan B, Smylie J (2015). First Peoples, second class treatment: The role of racism in the health and well-being of Indigenous peoples in Canada.

[40] Reading CL, Wien F. Health inequalities and the social determinants of Aboriginal peoples’ health. National Collaborating Centre for Aboriginal Health Prince George, BC; 2009.

[41] Walter M, Andersen C, Indigenous Statistics (2013). A Quantitative Research Methodology.

[42] Smylie J, Firestone M (2015). Back to the basics: Identifying and addressing underlying challenges in achieving high quality and relevant health statistics for Indigenous populations in Canada. Statistical Journal of the IAOS.

[43] Mosby I (2013). Administering colonial science: Nutrition research and human biomedical experimentation in Aboriginal communities and residential schools, 1942–1952. Social history.

[44] Harry D, Kaneche LaM (2006). Asserting tribal sovereignty over cultural property: moving towards protection of genetic material and indigenous knowledge. Seattle J Soc Just.

[45] Saltman RB, Ferroussier-Davis O (2000). The concept of stewardship in health policy. Bull World Health Organ.

[46] Veillard JHM, Brown AD, Barış E, Permanand G, Klazinga NS (2011). Health system stewardship of National Health Ministries in the WHO European region: concepts, functions and assessment framework. Health Policy.

[47] Wolfe S. The politics of reparations and apologies. Springer Science & Business Media; 2013.

[48] Truth and Reconciliation Commission of Canada (2015). Summary of the final report of the Truth and Reconciliation Commission of Canada.

[49] UN General Assembly. United Nations Declaration on the Rights of Indigenous Peoples. 2007.

[50] Girls NIiMaMIWa. Reclaiming Power and Place: The Final Report of the National Inquiry Into Missing and Murdered Indigenous Women and Girls. National Inquiry into Missing and Murdered Indigenous Women and Girls; 2019.

