# Supplementary material for: Selecting contextually appropriate performance indicators in a circumpolar context: a modified Delphi approach
Source: BMC Health Serv Res. 2021 May 30;21:530. doi: 10.1186/s12913-021-06485-2 (PMC8166122; doi:10.1186/s12913-021-06485-2)
Supplement: Supplementary file 1 — Additional file 1. Delphi Questionnaire Round 2. As outlined in the methods section, and is standard in a Delphi consensus process, the survey instruments used were designed for the purpose of this study. Survey item derivation is described above. This attached additional file includes a copy of the second round questionnaire as well as the associated preamble as it was distributed to Delphi panel members. The second round questionnaire has been included alone as it encompasses the both the contents of the first round questionnaire as well as the summary statistics generated in the first round and the additional indicators suggested by the Delphi panel in round 1. It thus provides a complete representation of the survey items distributed to panel members. [file 12913_2021_6485_MOESM1_ESM.docx]

**Circumpolar MCH Indicators (ROUND 2)**

Thank you so much for participating in ROUND 1 of this process!

A note about the indicators in ROUND 2: The group is close to reaching a consensus. Below you will find the same indicators you evaluated in ROUND 1. You will also find the mean and median group responses from ROUND 1. Some new items suggested by the panel have been added. As these represent concepts suggested by the panel, they are not accompanied by definitions from the literature.

A note about definitions: Based on your feedback, definitions of some indicators have been clarified or added to the alternative definitions provided in the attached info sheet. We realize that there are many different ways to define each indicator and that there are many possible ways to capture related phenomena. All of the definitions identified in the scoping review are included in the attached info sheet. The primary definition is that which was used most frequently in this body of international literature.

For ROUND2, please rate the indicators according to the same four criteria. You may use the ROUND 1 group responses to adjust your own responses.

**Criteria definitions: ​**

**Importance:** Level of concern of healthcare users or policy makers and the degree to which the indicator is susceptible to being influenced by the healthcare system

**Circumpolar Relevance:** Significance in the circumpolar context

**Validity:**Degree to which the indicator measures what it intends to measure

**Reliability:** Degree to which the indicator provides stable results across various populations, circumstances, and time points

​Please select your level of agreement with each statement according to the following scale.

1 - Strongly disagree

2 – Disagree

3 - Somewhat disagree

4 – Neutral

5 - Somewhat agree

6 – Agree

7 - Strongly Agree

If you do not know, or you prefer not to answer, please select N/A.

**Consent to participate in a research study**

Study Title:

Performance Measurement in a Circumpolar Context: Developing indicators for maternity care

Research Team:

Dr. Rebecca Rich

Resident physician, Department of Obstetrics and Gynecology, University of Toronto

M.Sc. Candidate, Institute of Health Policy Management and Evaluation, University of Toronto

120 Homewood Ave #116, Toronto, Ontario M4Y2J3 Canada

Tel: (416) 859-7424 (cell)

rebecca.rich@mail.utoronto.ca

Dr. Kellie Murphy

Associate Professor, Department of Obstetrics and Gynecology, University of Toronto

Associate Professor, Institute of Health Policy, Management and Evaluation, University of Toronto

Staff Physician, Mount Sinai Hospital

700 University Avenue, Room 3-918 Toronto, ON, M5G 1Z5 Canada

Tel: (416) 586-8570

Fax: (416) 586-4792

kmurphy@mtsinai.on.ca

Ms. Susan Chatwood

Assistant Professor, Dalla Lana School of Public Health, University of Toronto

Executive and scientific director, Institute for Circumpolar Health Research

PO Box 11050, Yellowknife, NT, X1A 3X7

Tel: (867) 873-9337

Fax: (867) 873-9338

susan.chatwood@ichr.ca

Dr. Jeremy Veillard,

Assistant Professor, Institute of Health Policy Management and Evaluation, University of Toronto

Strategic Policy Adviser, Health, Nutrition and Population Global Practice, The World Bank Group

1776 G Street NW Office 7-097

Washington, DC 20006 USA

Tel: (202) 790 2005 (cell)

jveillard@worldbank.org

You are being invited to participate in this research study because your peers have identified you as an expert in Northern maternity care, Northern health systems, or the development and evaluation of health systems performance indicators.

**About the Study:**

Assessment of performance in healthcare is a necessary component of a responsible health system. At present, maternity care systems in the circumpolar world are based on a model that treats health system responsiveness (the ability of the health system to meet the needs and expectations of the population it serves in accordance with the values of that population) and health outcomes as competing interests. However, in the context of low risk maternity care this conflict is not founded in evidence. A performance measurement strategy that recognizes a responsive health system as both a means to an end and an end in itself is a necessary step toward resolving this conflict. This project will use a modified Delphi consensus process to generate a set of contextually appropriate performance indicators for maternity care in circumpolar regions. The findings will help to inform the ongoing development of information systems in Northern Canada and contribute to the provision of safe, responsive, and culturally appropriate maternity care for Indigenous women.

**Your participation:**

Participation will include completion of a series of online surveys (two to three in total) over a period of 1-3 months. Each survey will take approximately 30-45 minutes to complete and will ask you to evaluate each indicator’s importance, relevance, validity, and reliability. Your participation is in this study is voluntary. You are may refuse to participate or withdraw your participation at any time without consequences. If you do not want to continue, you can simply leave the survey website prior to submitting your answers. You may also choose to skip any questions that you do not wish to or feel able to answer without consequences. Please note that the research study you are participating in may be reviewed for quality assurance to make sure that the required laws and guidelines are followed. If chosen, a representative of the Human Research Ethics Program (HREP) may access study-related data and/or consent materials as part of the review. All information accessed by the HREP will be upheld to the same level of confidentiality that has been stated by the research team.

**Access to information, confidentiality, and publication of results:**

Participation in the survey portion of the study will be anonymous and no information will be used to track individual participants’ survey answers. Thus, once you have submitted your survey responses, the data cannot be withdrawn. Anonymous survey responses will be collected through FluidSurvey servers and managed in accordance with their privacy and security policies. Of note, e-mail addresses of participants are safeguarded and not shared with outside sources. Following the data collection period, survey response data will be extracted and stored electronically on an encrypted laptop computer. Your contact information (email address), and data collected at the consensus meeting content will be stored in a similar fashion and will be accessible only to the research team. Data will be stored for five years after which time it will be deleted. Quotations from your responses to survey questions may be used for academic presentation or publication. You will also receive final project report at its conclusion.

**Risks and benefits:**

By participating in this study you will be contributing to a performance measurement strategy that reflects the values and context unique to maternity care in Northern regions. There are no risks to you for participating in this study. No consequences will occur if you choose not to participate.

**Contact Information:**

Should you have further questions you may contact any of the researchers (contact information above). If you have questions about your rights as a participant in research, please contact the Research Oversight and Compliance Office - Human Research Ethics Program at ethics.review@utoronto.ca or 416-946-3273

**By beginning the survey, you acknowledge that you have read this information, have had your questions answered, and freely agree to participate in this study. You may print or retain a copy of this form for your records.**

**DETERMINANTS OF HEALTH**

The following indicators represent non-healthcare factors that determine health. These can be health behaviours, personal resources, socioeconomic, environmental, or physical factors.

**Teenage birth rate**

Number of births per 1000 women ages 15-19

|  | 1 | 2 | 3 | 4 | 5 | 6 | 7 | N/A |
| --- | --- | --- | --- | --- | --- | --- | --- | --- |
| This indicator is important. [mean=6.07, median=6, SD=1.21) |  |  |  |  |  |  |  |  |
| This indicator is relevant to the circumpolar context. [mean=6.36, median=7, SD=1.08) |  |  |  |  |  |  |  |  |
| This indicator is valid. [mean=6.28, median=7, SD=1.07) |  |  |  |  |  |  |  |  |
| This indicator is reliable. [mean=6.54, median=7, SD=0.66) |  |  |  |  |  |  |  |  |

**Advanced maternal age**

Proportion of live births to women 35 or older

|  | 1 | 2 | 3 | 4 | 5 | 6 | 7 | N/A |
| --- | --- | --- | --- | --- | --- | --- | --- | --- |
| This indicator is important. [mean=4.86, median=5, SD=1.46) |  |  |  |  |  |  |  |  |
| This indicator is relevant to the circumpolar context. [mean=4.35, median=4, SD=1.45) |  |  |  |  |  |  |  |  |
| This indicator is valid.[mean=6.00, median=6, SD=1.08) |  |  |  |  |  |  |  |  |
| This indicator is reliable. [mean=6.15, median=6, SD=0.90) |  |  |  |  |  |  |  |  |

**Maternal Body Mass Index (BMI)**

Distribution of mother’s pre-pregnancy body mass index (BMI)

|  | 1 | 2 | 3 | 4 | 5 | 6 | 7 | N/A |
| --- | --- | --- | --- | --- | --- | --- | --- | --- |
| This indicator is important. [mean=5.71, median=6, SD=1.38) |  |  |  |  |  |  |  |  |
| This indicator is relevant to the circumpolar context. [mean=5.71, median=6.5, SD=1.64) |  |  |  |  |  |  |  |  |
| This indicator is valid. [mean=5.30, median=5, SD=1.75) |  |  |  |  |  |  |  |  |
| This indicator is reliable. [mean=4.85, median=5, SD=1.82) |  |  |  |  |  |  |  |  |

**Maternal education level**

Proportion of pregnancies to mothers with education level less than grade 12

|  | 1 | 2 | 3 | 4 | 5 | 6 | 7 | N/A |
| --- | --- | --- | --- | --- | --- | --- | --- | --- |
| This indicator is important. [mean=6.31, median=7, SD=1.03) |  |  |  |  |  |  |  |  |
| This indicator is relevant to the circumpolar context. [mean=6.43, median=7, SD=0.76) |  |  |  |  |  |  |  |  |
| This indicator is valid. [mean=6.15, median=6, SD=0.69) |  |  |  |  |  |  |  |  |
| This indicator is reliable. [mean=5.64, median=6, SD=1.22) |  |  |  |  |  |  |  |  |

**Domestic violence**

Proportion of pregnant women reporting physical or sexual abuse in the past two years

|  | 1 | 2 | 3 | 4 | 5 | 6 | 7 | N/A |
| --- | --- | --- | --- | --- | --- | --- | --- | --- |
| This indicator is important. [mean=6.53, median=7, SD=0.97] |  |  |  |  |  |  |  |  |
| This indicator is relevant to the circumpolar context. [mean=6.64, median=7, SD=0.63] |  |  |  |  |  |  |  |  |
| This indicator is valid. [mean=5.58, median=5.5, SD=1.16] |  |  |  |  |  |  |  |  |
| This indicator is reliable. [mean=4.14, median=4, SD=1.66] |  |  |  |  |  |  |  |  |

**Smoking during pregnancy**

Proportion of women who smoked during pregnancy

|  | 1 | 2 | 3 | 4 | 5 | 6 | 7 | N/A |
| --- | --- | --- | --- | --- | --- | --- | --- | --- |
| This indicator is important. [mean=6.92, median=7, SD=0.23] |  |  |  |  |  |  |  |  |
| This indicator is relevant to the circumpolar context. [mean=6.92, median=7, SD=0.27] |  |  |  |  |  |  |  |  |
| This indicator is valid. [mean=5.93, median6=, SD=1.27] |  |  |  |  |  |  |  |  |
| This indicator is reliable. [mean=5.43, median=6, SD=1.87] |  |  |  |  |  |  |  |  |

**Use of illicit drugs during pregnancy**

Proportion of women who report using illicit substances during pregnancy

|  | 1 | 2 | 3 | 4 | 5 | 6 | 7 | N/A |
| --- | --- | --- | --- | --- | --- | --- | --- | --- |
| This indicator is important. [mean=6.86, median=7, SD=0.36] |  |  |  |  |  |  |  |  |
| This indicator is relevant to the circumpolar context. [mean=6.57, median=7, SD=0.65] |  |  |  |  |  |  |  |  |
| This indicator is valid. [mean=5.23, median=5, SD=1.30] |  |  |  |  |  |  |  |  |
| This indicator is reliable. [mean=4.07, median4=, SD=1.64] |  |  |  |  |  |  |  |  |

**Use of alcohol during pregnancy**

Proportion of mothers reporting alcohol use in pregnancy

|  | 1 | 2 | 3 | 4 | 5 | 6 | 7 | N/A |
| --- | --- | --- | --- | --- | --- | --- | --- | --- |
| This indicator is important. [mean=6.71, median=7, SD=0.61] |  |  |  |  |  |  |  |  |
| This indicator is relevant to the circumpolar context. [mean=6.64, median=7, SD=0.74] |  |  |  |  |  |  |  |  |
| This indicator is valid. [mean=5.61, median=5, SD=1.33] |  |  |  |  |  |  |  |  |
| This indicator is reliable. [mean=4.15, median=4, SD=1.63] |  |  |  |  |  |  |  |  |

**Exposure to environmental contaminants**

Persistent organic pollutant (POP) concentration in breast milk

|  | 1 | 2 | 3 | 4 | 5 | 6 | 7 | N/A |
| --- | --- | --- | --- | --- | --- | --- | --- | --- |
| This indicator is important. [mean=5.67, median=6, SD=1.43] |  |  |  |  |  |  |  |  |
| This indicator is relevant to the circumpolar context. [mean=5.77, median=6, SD=1.30] |  |  |  |  |  |  |  |  |
| This indicator is valid. [mean=5.08, median=6, SD=1.78] |  |  |  |  |  |  |  |  |
| This indicator is reliable. [mean=5.50, median=6, SD=1.98] |  |  |  |  |  |  |  |  |

**Stressors during pregnancy**

Proportion of women who reported experiencing two or more stressors during pregnancy

|  | 1 | 2 | 3 | 4 | 5 | 6 | 7 | N/A |
| --- | --- | --- | --- | --- | --- | --- | --- | --- |
| This indicator is important. [mean=5.30, median=6, SD=1.60] |  |  |  |  |  |  |  |  |
| This indicator is relevant to the circumpolar context. [mean=5.71, median=6, SD=1.33] |  |  |  |  |  |  |  |  |
| This indicator is valid. [mean=4.15, median=4, SD=1.86] |  |  |  |  |  |  |  |  |
| This indicator is reliable. [mean=3.85, median=3, SD=1.91] |  |  |  |  |  |  |  |  |

**Breastfeeding practices**

Proportion of newborns that were exclusively breastfed through the first 48 hours of life

|  | 1 | 2 | 3 | 4 | 5 | 6 | 7 | N/A |
| --- | --- | --- | --- | --- | --- | --- | --- | --- |
| This indicator is important. [mean=6.54, median=7, SD=0.66] |  |  |  |  |  |  |  |  |
| This indicator is relevant to the circumpolar context. [mean=6.77, median=7, SD=0.44] |  |  |  |  |  |  |  |  |
| This indicator is valid. [mean=6.31, median=6, SD=0.75] |  |  |  |  |  |  |  |  |
| This indicator is reliable. [mean=5.77, median=6, SD=1.24] |  |  |  |  |  |  |  |  |

**Involvement of child and family services or similar organization**

Proportion of births to women who have had contact with the Department of Child safety

|  | 1 | 2 | 3 | 4 | 5 | 6 | 7 | N/A |
| --- | --- | --- | --- | --- | --- | --- | --- | --- |
| This indicator is important. [mean=5.69, median=6, SD=1.03] |  |  |  |  |  |  |  |  |
| This indicator is relevant to the circumpolar context. [mean=6.08, median=6, SD=0.67] |  |  |  |  |  |  |  |  |
| This indicator is valid. [mean=5.00, median=5, SD=1.41] |  |  |  |  |  |  |  |  |
| This indicator is reliable. [mean=4.75, median=5, SD=1.60] |  |  |  |  |  |  |  |  |

**Maternal history of adverse childhood experiences**

|  | 1 | 2 | 3 | 4 | 5 | 6 | 7 | N/A |
| --- | --- | --- | --- | --- | --- | --- | --- | --- |
| This indicator is important. |  |  |  |  |  |  |  |  |
| This indicator is relevant to the circumpolar context. |  |  |  |  |  |  |  |  |

**Family income or income distribution**

|  | 1 | 2 | 3 | 4 | 5 | 6 | 7 | N/A |
| --- | --- | --- | --- | --- | --- | --- | --- | --- |
| This indicator is important. |  |  |  |  |  |  |  |  |
| This indicator is relevant to the circumpolar context. |  |  |  |  |  |  |  |  |

**Food insecurity**

|  | 1 | 2 | 3 | 4 | 5 | 6 | 7 | N/A |
| --- | --- | --- | --- | --- | --- | --- | --- | --- |
| This indicator is important. |  |  |  |  |  |  |  |  |
| This indicator is relevant to the circumpolar context. |  |  |  |  |  |  |  |  |

**Level of maternal physical activity**

|  | 1 | 2 | 3 | 4 | 5 | 6 | 7 | N/A |
| --- | --- | --- | --- | --- | --- | --- | --- | --- |
| This indicator is important. |  |  |  |  |  |  |  |  |
| This indicator is relevant to the circumpolar context. |  |  |  |  |  |  |  |  |

**Maternal oral health**

|  | 1 | 2 | 3 | 4 | 5 | 6 | 7 | N/A |
| --- | --- | --- | --- | --- | --- | --- | --- | --- |
| This indicator is important. |  |  |  |  |  |  |  |  |
| This indicator is relevant to the circumpolar context. |  |  |  |  |  |  |  |  |

**Maternal smokeless tobacco use**

|  | 1 | 2 | 3 | 4 | 5 | 6 | 7 | N/A |
| --- | --- | --- | --- | --- | --- | --- | --- | --- |
| This indicator is important. |  |  |  |  |  |  |  |  |
| This indicator is relevant to the circumpolar context. |  |  |  |  |  |  |  |  |

**Maternal housing (experiences of overcrowding or being inadequately or under-housed)**

|  | 1 | 2 | 3 | 4 | 5 | 6 | 7 | N/A |
| --- | --- | --- | --- | --- | --- | --- | --- | --- |
| This indicator is important. |  |  |  |  |  |  |  |  |
| This indicator is relevant to the circumpolar context. |  |  |  |  |  |  |  |  |

**If you have any additional comments, please include them here.**

**EFFECTIVENESS**

The following indicators represent indicators of health system effectiveness. Effectiveness refers to the degree of achieving desirable outcomes, given the correct provision of evidence-based healthcare services to all who could benefit, not to those who would not benefit (Arah et al. 2003).

**Anemia**

Proportion of women diagnosed with anemia during pregnancy

|  | 1 | 2 | 3 | 4 | 5 | 6 | 7 | N/A |
| --- | --- | --- | --- | --- | --- | --- | --- | --- |
| This indicator is important. [mean=6.57, median=7, SD=0.65] |  |  |  |  |  |  |  |  |
| This indicator is relevant to the circumpolar context. [mean=,6.71 median=7, SD=0.47] |  |  |  |  |  |  |  |  |
| This indicator is valid. [mean=6.18, median=6, SD=0.98] |  |  |  |  |  |  |  |  |
| This indicator is reliable. [mean=6.08, median=6, SD=1.23] |  |  |  |  |  |  |  |  |

**Eclampsia**

Rate of eclampsia (per 1000 births)

|  | 1 | 2 | 3 | 4 | 5 | 6 | 7 | N/A |
| --- | --- | --- | --- | --- | --- | --- | --- | --- |
| This indicator is important. [mean=5.79, median=6.5, SD=1.76] |  |  |  |  |  |  |  |  |
| This indicator is relevant to the circumpolar context. [mean=5.86, median=6.5, SD=1.66] |  |  |  |  |  |  |  |  |
| This indicator is valid. [mean=5.92, median=6, SD=1.38] |  |  |  |  |  |  |  |  |
| This indicator is reliable. [mean=5.50, median=6, SD=1.31] |  |  |  |  |  |  |  |  |

**Diabetes in pregnancy**

Proportion of women diagnosed with gestational diabetes (per 1000 births)

|  | 1 | 2 | 3 | 4 | 5 | 6 | 7 | N/A |
| --- | --- | --- | --- | --- | --- | --- | --- | --- |
| This indicator is important. [mean=6.57, median=7, SD=0.65] |  |  |  |  |  |  |  |  |
| This indicator is relevant to the circumpolar context. [mean=6.35, median=6.5, SD=0.74] |  |  |  |  |  |  |  |  |
| This indicator is valid. [mean=5.85, median=6, SD=1.46] |  |  |  |  |  |  |  |  |
| This indicator is reliable. [mean=5.62, median=6, SD=1.45] |  |  |  |  |  |  |  |  |

**Folic acid supplementation**

Proportion of women who reported taking folic acid supplementation preconception

|  | 1 | 2 | 3 | 4 | 5 | 6 | 7 | N/A |
| --- | --- | --- | --- | --- | --- | --- | --- | --- |
| This indicator is important. [mean=6.14, median=6, SD=0.84] |  |  |  |  |  |  |  |  |
| This indicator is relevant to the circumpolar context. [mean=6.14, median=6, SD=0.86] |  |  |  |  |  |  |  |  |
| This indicator is valid. [mean=5.33, median=5.5, SD=1.23] |  |  |  |  |  |  |  |  |
| This indicator is reliable. [mean=4.2, median=4, SD=1.59] |  |  |  |  |  |  |  |  |

**HIV testing**

Proportion of women who reported having HIV testing in pregnancy

|  | 1 | 2 | 3 | 4 | 5 | 6 | 7 | N/A |
| --- | --- | --- | --- | --- | --- | --- | --- | --- |
| This indicator is important. [mean=5.36, median=5.5, SD=1.39] |  |  |  |  |  |  |  |  |
| This indicator is relevant to the circumpolar context. [mean=5.00, median=5, SD=1.58] |  |  |  |  |  |  |  |  |
| This indicator is valid. [mean=6.09, median=6, SD=0.94] |  |  |  |  |  |  |  |  |
| This indicator is reliable. [mean=5.91, median=6, SD=1.14] |  |  |  |  |  |  |  |  |

**Spontaneous abortions**

Proportion of all pregnancies which end in spontaneous abortion

|  | 1 | 2 | 3 | 4 | 5 | 6 | 7 | N/A |
| --- | --- | --- | --- | --- | --- | --- | --- | --- |
| This indicator is important. [mean=5.42, median=5.5, SD=1.16] |  |  |  |  |  |  |  |  |
| This indicator is relevant to the circumpolar context. [mean=5.31, median=6, SD=1.25] |  |  |  |  |  |  |  |  |
| This indicator is valid. [mean=4.83, median=5, SD=1.40] |  |  |  |  |  |  |  |  |
| This indicator is reliable. [mean=4.41, median=5, SD=1.56] |  |  |  |  |  |  |  |  |

**Stillbirths**

Fetal deaths (after 28 weeks GA, or greater than or equal to 1000g where GA is not available) per 1000 births

|  | 1 | 2 | 3 | 4 | 5 | 6 | 7 | N/A |
| --- | --- | --- | --- | --- | --- | --- | --- | --- |
| This indicator is important. [mean=6.21, median=6.5, SD=1.12] |  |  |  |  |  |  |  |  |
| This indicator is relevant to the circumpolar context. [mean=6.07, median=6, SD=1.07] |  |  |  |  |  |  |  |  |
| This indicator is valid. [mean=6.08, median=7, SD=1.44] |  |  |  |  |  |  |  |  |
| This indicator is reliable. [mean=6.00, median=6, SD=1.41] |  |  |  |  |  |  |  |  |

**Perinatal death**

Perinatal deaths (stillbirths after 28 weeks plus neonatal deaths within the first 7 days of life) per 1000 births

|  | 1 | 2 | 3 | 4 | 5 | 6 | 7 | N/A |
| --- | --- | --- | --- | --- | --- | --- | --- | --- |
| This indicator is important. [mean=6.64, median=7, SD=0.63] |  |  |  |  |  |  |  |  |
| This indicator is relevant to the circumpolar context. [mean=6.50, median=7, SD=0.65] |  |  |  |  |  |  |  |  |
| This indicator is valid. [mean=6.46, median=7, SD=.066] |  |  |  |  |  |  |  |  |
| This indicator is reliable. [mean=6.38, median=6, SD=0.42] |  |  |  |  |  |  |  |  |

**Preterm births**

Proportion of all births at less than 37 weeks GA

|  | 1 | 2 | 3 | 4 | 5 | 6 | 7 | N/A |
| --- | --- | --- | --- | --- | --- | --- | --- | --- |
| This indicator is important. [mean=6.79, median=7, SD=0.43] |  |  |  |  |  |  |  |  |
| This indicator is relevant to the circumpolar context. [mean=6.79, median=7, SD=0.43] |  |  |  |  |  |  |  |  |
| This indicator is valid. [mean=6.30, median=7, SD=]1.11 |  |  |  |  |  |  |  |  |
| This indicator is reliable. [mean=6.23, median=6, SD=1.09] |  |  |  |  |  |  |  |  |

**Post term births**

Proportion of all births at greater than 42 weeks GA

|  | 1 | 2 | 3 | 4 | 5 | 6 | 7 | N/A |
| --- | --- | --- | --- | --- | --- | --- | --- | --- |
| This indicator is important. [mean=5.28, median=6, SD=1.64] |  |  |  |  |  |  |  |  |
| This indicator is relevant to the circumpolar context. [mean=5.07, median=5, SD=1.21] |  |  |  |  |  |  |  |  |
| This indicator is valid. [mean=5.61, median=6, SD=0.87] |  |  |  |  |  |  |  |  |
| This indicator is reliable. [mean=5.53, median=6, SD=1.13] |  |  |  |  |  |  |  |  |

**Induction and augmentation of labour**

Mode of onset of labour (spontaneous vs. induced labour) per 100 live births and stillbirths

|  | 1 | 2 | 3 | 4 | 5 | 6 | 7 | N/A |
| --- | --- | --- | --- | --- | --- | --- | --- | --- |
| This indicator is important. [mean=5.64, median=6, SD=1.22] |  |  |  |  |  |  |  |  |
| This indicator is relevant to the circumpolar context. [mean=5.29, median=5, SD=1.27] |  |  |  |  |  |  |  |  |
| This indicator is valid. [mean=5.85, median=6, SD=1.24] |  |  |  |  |  |  |  |  |
| This indicator is reliable. [mean=5.75, median=6, SD=1.14] |  |  |  |  |  |  |  |  |

**VBAC (after single previous C/S)**

Proportion of multiparous mothers who have had one previous caesarean, whose current method of birth was either an instrumental or non-instrumental vaginal delivery

|  | 1 | 2 | 3 | 4 | 5 | 6 | 7 | N/A |
| --- | --- | --- | --- | --- | --- | --- | --- | --- |
| This indicator is important. [mean=5.57, median=6, SD=1.34] |  |  |  |  |  |  |  |  |
| This indicator is relevant to the circumpolar context. [mean=5.21, median=6, SD=1.53] |  |  |  |  |  |  |  |  |
| This indicator is valid. [mean=6.08, median=6, SD=1.04] |  |  |  |  |  |  |  |  |
| This indicator is reliable. [mean=6.08, median=6, SD=1.04] |  |  |  |  |  |  |  |  |

**Instrumental vaginal deliveries**

Percentage of all births by instrumental vaginal delivery (vacuum/forceps)

|  | 1 | 2 | 3 | 4 | 5 | 6 | 7 | N/A |
| --- | --- | --- | --- | --- | --- | --- | --- | --- |
| This indicator is important. [mean=5.62, median=6, SD=1.39] |  |  |  |  |  |  |  |  |
| This indicator is relevant to the circumpolar context. [mean=5.23, median=6, SD=1.36] |  |  |  |  |  |  |  |  |
| This indicator is valid. [mean=6.17, median=6, SD=1.11] |  |  |  |  |  |  |  |  |
| This indicator is reliable. [mean=5.92, median=6, SD=1.44] |  |  |  |  |  |  |  |  |

**Caesarean sections**

Percentage of all births (live and stillbirths) by caesarean section

|  | 1 | 2 | 3 | 4 | 5 | 6 | 7 | N/A |  |
| --- | --- | --- | --- | --- | --- | --- | --- | --- | --- |
| This indicator is important. [mean=6.43, median=6.5, SD=0.65] |  |  |  |  |  |  |  |  |  |
| This indicator is relevant to the circumpolar context. [mean=6.07, median=6, SD=1.07] |  |  |  |  |  |  |  |  |  |
| This indicator is valid. [mean=6.38, median=7, SD=0.87] |  |  |  |  |  |  |  |  |  |
| This indicator is reliable. [mean6.53=, median=7, SD=0.52] |  |  |  |  |  |  |  |  |  |

**Maternal mortality**

Maternal mortality ratio (MMR): The number of maternal deaths during a given time period per 100,000 live births during the same time-period

|  | 1 | 2 | 3 | 4 | 5 | 6 | 7 | N/A |
| --- | --- | --- | --- | --- | --- | --- | --- | --- |
| This indicator is important. [mean=6.57, median7=, SD=0.65] |  |  |  |  |  |  |  |  |
| This indicator is relevant to the circumpolar context. [mean=6.5, median=7, SD=0.76] |  |  |  |  |  |  |  |  |
| This indicator is valid. [mean=6.62, median=7, SD=0.51] |  |  |  |  |  |  |  |  |
| This indicator is reliable. [mean=6.62, median=7, SD=0.51] |  |  |  |  |  |  |  |  |

**Postpartum hemorrhage**

Proportion of women who had an estimated blood loss of >1000 mL at delivery

|  | 1 | 2 | 3 | 4 | 5 | 6 | 7 | N/A |
| --- | --- | --- | --- | --- | --- | --- | --- | --- |
| This indicator is important. [mean=6.46, median=7, SD=0.66] |  |  |  |  |  |  |  |  |
| This indicator is relevant to the circumpolar context. [mean=6.23, median=6, SD=0.83] |  |  |  |  |  |  |  |  |
| This indicator is valid. [mean=5.83, median=6, SD=1.47] |  |  |  |  |  |  |  |  |
| This indicator is reliable. [mean=5.25, median=5.5, SD=1.54] |  |  |  |  |  |  |  |  |

**Postpartum depression**

Proportion of women who scored > 14 on the Edinburgh Depression scale

|  | 1 | 2 | 3 | 4 | 5 | 6 | 7 | N/A |
| --- | --- | --- | --- | --- | --- | --- | --- | --- |
| This indicator is important. [mean=6.4, median=7, SD=0.79] |  |  |  |  |  |  |  |  |
| This indicator is relevant to the circumpolar context. [mean=6.33, median=6.5, SD=0.78] |  |  |  |  |  |  |  |  |
| This indicator is valid. [mean=4.67, median=5, SD=2.02] |  |  |  |  |  |  |  |  |
| This indicator is reliable. [mean=3.91, median=3, SD=2.12] |  |  |  |  |  |  |  |  |

**Postpartum infections**

Proportion of women who suffered a surgical site infection within 30 days of caesarean section

|  | 1 | 2 | 3 | 4 | 5 | 6 | 7 | N/A |
| --- | --- | --- | --- | --- | --- | --- | --- | --- |
| This indicator is important. [mean=5.92, median=6, SD=0.95] |  |  |  |  |  |  |  |  |
| This indicator is relevant to the circumpolar context. [mean=5.61, median=6, SD=1.12] |  |  |  |  |  |  |  |  |
| This indicator is valid. [mean=5.5, median=6, SD=1.24] |  |  |  |  |  |  |  |  |
| This indicator is reliable. [mean=5.00, median=6, SD=1.60] |  |  |  |  |  |  |  |  |

**Maternal readmissions to hospital**

Risk adjusted rate of readmission to hospital after an admission for obstetric indications

|  | 1 | 2 | 3 | 4 | 5 | 6 | 7 | N/A |
| --- | --- | --- | --- | --- | --- | --- | --- | --- |
| This indicator is important. [mean=5.71, median=6, SD=1.14] |  |  |  |  |  |  |  |  |
| This indicator is relevant to the circumpolar context. [mean=5.57, median=6, SD=1.22] |  |  |  |  |  |  |  |  |
| This indicator is valid. [mean=5.61, median=6, SD=1.26] |  |  |  |  |  |  |  |  |
| This indicator is reliable. [mean=5.54, median=6, SD=1.33] |  |  |  |  |  |  |  |  |

**Postpartum contraception**

Proportion of women using birth control postpartum

|  | 1 | 2 | 3 | 4 | 5 | 6 | 7 | N/A |
| --- | --- | --- | --- | --- | --- | --- | --- | --- |
| This indicator is important. [mean=6.35, median=7, SD=0.93] |  |  |  |  |  |  |  |  |
| This indicator is relevant to the circumpolar context. [mean=6.28, median=7, SD=1.07] |  |  |  |  |  |  |  |  |
| This indicator is valid. [mean=5.58, median=6, SD=1.08] |  |  |  |  |  |  |  |  |
| This indicator is reliable. [mean=4.67, median=4.5, SD=1.56] |  |  |  |  |  |  |  |  |

**Neonatal mortality**

Neonatal death (from birth to 28 days of life) per 1000 live births occurring at or after 24 weeks GA

|  | 1 | 2 | 3 | 4 | 5 | 6 | 7 | N/A |
| --- | --- | --- | --- | --- | --- | --- | --- | --- |
| This indicator is important. [mean=6.86, median7=, SD=0.36] |  |  |  |  |  |  |  |  |
| This indicator is relevant to the circumpolar context. [mean=6.86, median=7, SD=0.36] |  |  |  |  |  |  |  |  |
| This indicator is valid. [mean=6.61, median=7, SD=0.51] |  |  |  |  |  |  |  |  |
| This indicator is reliable. [mean=6.54, median=7, SD=0.52] |  |  |  |  |  |  |  |  |

**Congenital anomalies**

Prevalence of major congenital anomalies

|  | 1 | 2 | 3 | 4 | 5 | 6 | 7 | N/A |
| --- | --- | --- | --- | --- | --- | --- | --- | --- |
| This indicator is important. [mean=6.14, median=6.5, SD=1.17] |  |  |  |  |  |  |  |  |
| This indicator is relevant to the circumpolar context. [mean=6.14, median=6.5, SD=1.17] |  |  |  |  |  |  |  |  |
| This indicator is valid. [mean=6.00, median=6, SD=1.29] |  |  |  |  |  |  |  |  |
| This indicator is reliable. [mean=5.54, median=6, SD=1.66] |  |  |  |  |  |  |  |  |

**Small for gestational age Infants**

Proportion of live born singleton newborns weighing <10th %ile for GA

|  | 1 | 2 | 3 | 4 | 5 | 6 | 7 | N/A |
| --- | --- | --- | --- | --- | --- | --- | --- | --- |
| This indicator is important. [mean=6.5, median=7, SD=0.65] |  |  |  |  |  |  |  |  |
| This indicator is relevant to the circumpolar context. [mean=6.35, median=7, SD=1.34] |  |  |  |  |  |  |  |  |
| This indicator is valid. [mean=6.38, median=6, SD=0.51] |  |  |  |  |  |  |  |  |
| This indicator is reliable. [mean=5.92, median=6, SD=1.19] |  |  |  |  |  |  |  |  |

**Low birth weight infants**

Proportion of live born infants weighing <2500g

|  | 1 | 2 | 3 | 4 | 5 | 6 | 7 | N/A |
| --- | --- | --- | --- | --- | --- | --- | --- | --- |
| This indicator is important. [mean=6.43, median=6.5, SD=0.65] |  |  |  |  |  |  |  |  |
| This indicator is relevant to the circumpolar context. [mean=6.21, median=6.5, SD=1.31] |  |  |  |  |  |  |  |  |
| This indicator is valid. [mean=6.54, median=7, SD=0.52] |  |  |  |  |  |  |  |  |
| This indicator is reliable. [mean=6.54, median=7, SD=0.52] |  |  |  |  |  |  |  |  |

**Large for gestational age infants**

Proportion of live born singleton newborns • Proportion of live born singleton newborns weighing >90th %ile for GA

|  | 1 | 2 | 3 | 4 | 5 | 6 | 7 | N/A |
| --- | --- | --- | --- | --- | --- | --- | --- | --- |
| This indicator is important. [mean=6.00, median=6, SD=0.97] |  |  |  |  |  |  |  |  |
| This indicator is relevant to the circumpolar context. [mean=5.71, median=6, SD=0.91] |  |  |  |  |  |  |  |  |
| This indicator is valid. [mean=5.92, median=6, SD=0.76] |  |  |  |  |  |  |  |  |
| This indicator is reliable. [mean=5.69, median=6, SD=1.37] |  |  |  |  |  |  |  |  |

**Five minute Apgar score**

Proportion of newborns with 5 minute Apgar score < 7

|  | 1 | 2 | 3 | 4 | 5 | 6 | 7 | N/A |
| --- | --- | --- | --- | --- | --- | --- | --- | --- |
| This indicator is important. [mean=5.71, median=6, SD=1.44] |  |  |  |  |  |  |  |  |
| This indicator is relevant to the circumpolar context. [mean=5.71, median=6, SD=1.33] |  |  |  |  |  |  |  |  |
| This indicator is valid. [mean=5.46, median=6, SD=1.45] |  |  |  |  |  |  |  |  |
| This indicator is reliable. [mean=5.15, median=5, SD=1.72] |  |  |  |  |  |  |  |  |

**NICU admissions**

Proportion of newborns requiring admission to a neonatal intensive care unit (NICU)

|  | 1 | 2 | 3 | 4 | 5 | 6 | 7 | N/A |
| --- | --- | --- | --- | --- | --- | --- | --- | --- |
| This indicator is important. [mean=6.08, median6=, SD=1.12] |  |  |  |  |  |  |  |  |
| This indicator is relevant to the circumpolar context. [mean=5.71, median=6, SD=1.44] |  |  |  |  |  |  |  |  |
| This indicator is valid. [mean=6.23, median=6, SD=1.09] |  |  |  |  |  |  |  |  |
| This indicator is reliable. [mean=5.62, median=6, SD=1.56] |  |  |  |  |  |  |  |  |

**Neonatal readmission to hospital**

Rate of neonatal hospital readmission after discharge following birth

|  | 1 | 2 | 3 | 4 | 5 | 6 | 7 | N/A |
| --- | --- | --- | --- | --- | --- | --- | --- | --- |
| This indicator is important. [mean=5.86, median=6, SD=1.10] |  |  |  |  |  |  |  |  |
| This indicator is relevant to the circumpolar context. [mean=5.86, median=6, SD=1.10] |  |  |  |  |  |  |  |  |
| This indicator is valid. [mean=6.08, median=6, SD=1.04] |  |  |  |  |  |  |  |  |
| This indicator is reliable. [mean=5.92, median=6, SD=1.32] |  |  |  |  |  |  |  |  |

**Vitamin D supplementation**

|  | 1 | 2 | 3 | 4 | 5 | 6 | 7 | N/A |
| --- | --- | --- | --- | --- | --- | --- | --- | --- |
| This indicator is important. |  |  |  |  |  |  |  |  |
| This indicator is relevant to the circumpolar context. |  |  |  |  |  |  |  |  |

**Prenatal vitamin use**

|  | 1 | 2 | 3 | 4 | 5 | 6 | 7 | N/A |
| --- | --- | --- | --- | --- | --- | --- | --- | --- |
| This indicator is important. |  |  |  |  |  |  |  |  |
| This indicator is relevant to the circumpolar context. |  |  |  |  |  |  |  |  |

**Screening for gestational diabetes (GDM)**

|  | 1 | 2 | 3 | 4 | 5 | 6 | 7 | N/A |
| --- | --- | --- | --- | --- | --- | --- | --- | --- |
| This indicator is important. |  |  |  |  |  |  |  |  |
| This indicator is relevant to the circumpolar context. |  |  |  |  |  |  |  |  |

**Rate of unintended pregnancies**

|  | 1 | 2 | 3 | 4 | 5 | 6 | 7 | N/A |
| --- | --- | --- | --- | --- | --- | --- | --- | --- |
| This indicator is important. |  |  |  |  |  |  |  |  |
| This indicator is relevant to the circumpolar context. |  |  |  |  |  |  |  |  |

**If you have any additional comments, please include them here.**

**SAFETY**

The following indicators represent safety. Safety refers to the degree to which healthcare avoids and prevents adverse outcomes that are a result of the healthcare itself (National Patient Safety Foundation, 2000).

**Births without obstetric intervention**

Proportion of births occurring without obstetric intervention

|  | 1 | 2 | 3 | 4 | 5 | 6 | 7 | N/A |
| --- | --- | --- | --- | --- | --- | --- | --- | --- |
| This indicator is important. [mean=6.33, median=6.5, SD=0.89] |  |  |  |  |  |  |  |  |
| This indicator is relevant to the circumpolar context. [mean=6.12, median=6, SD=0.94] |  |  |  |  |  |  |  |  |
| This indicator is valid. [mean=5.9, median=6, SD=0.99] |  |  |  |  |  |  |  |  |
| This indicator is reliable. [mean=5.27, median=6, SD=1.62] |  |  |  |  |  |  |  |  |

**Perineal trauma**

Proportion of women delivering vaginally who had a 3rd or 4th degree tear

|  | 1 | 2 | 3 | 4 | 5 | 6 | 7 | N/A |
| --- | --- | --- | --- | --- | --- | --- | --- | --- |
| This indicator is important. [mean=5.58, median=6, SD=1.16] |  |  |  |  |  |  |  |  |
| This indicator is relevant to the circumpolar context. [mean=5.5, median=6, SD=1.09] |  |  |  |  |  |  |  |  |
| This indicator is valid. [mean=6.09, median=6, SD=0.54] |  |  |  |  |  |  |  |  |
| This indicator is reliable. [mean=5.91, median=6, SD=.70] |  |  |  |  |  |  |  |  |

**Transfers for obstetrical indications (antepartum, intrapartum, postpartum)**

|  | 1 | 2 | 3 | 4 | 5 | 6 | 7 | N/A |
| --- | --- | --- | --- | --- | --- | --- | --- | --- |
| This indicator is important. |  |  |  |  |  |  |  |  |
| This indicator is relevant to the circumpolar context. |  |  |  |  |  |  |  |  |

**Unplanned births in the community (by term/preterm status)**

|  | 1 | 2 | 3 | 4 | 5 | 6 | 7 | N/A |
| --- | --- | --- | --- | --- | --- | --- | --- | --- |
| This indicator is important. |  |  |  |  |  |  |  |  |
| This indicator is relevant to the circumpolar context. |  |  |  |  |  |  |  |  |

**If you have any additional comments, please include them here.**

**RESPONSIVENESS**

The following indicators refer to health system responsiveness. Responsiveness refers to the ability of the health system to "meet the population's legitimate expecations regarding their interaction with the health system, apart from expectations for improvements in health or wealth" (WHO 2000).

**Characteristics of care providers**

Proportion of Aboriginal people in the health workforce

|  | 1 | 2 | 3 | 4 | 5 | 6 | 7 | N/A |
| --- | --- | --- | --- | --- | --- | --- | --- | --- |
| This indicator is important. [mean=6.21, median=7, SD=1.42] |  |  |  |  |  |  |  |  |
| This indicator is relevant to the circumpolar context. [mean=6.5, median=7, SD=1.09] |  |  |  |  |  |  |  |  |
| This indicator is valid. [mean=5.77, median=6, SD=1.36] |  |  |  |  |  |  |  |  |
| This indicator is reliable. [mean=5.3, median=6, SD=1.37] |  |  |  |  |  |  |  |  |

**Cultural competency**

Proportion of health care services with cultural safety policies or processes in place

|  | 1 | 2 | 3 | 4 | 5 | 6 | 7 | N/A |
| --- | --- | --- | --- | --- | --- | --- | --- | --- |
| This indicator is important. [mean=6.31, median=7, SD=1.44] |  |  |  |  |  |  |  |  |
| This indicator is relevant to the circumpolar context. [mean=6.46, median=7, SD=1.20] |  |  |  |  |  |  |  |  |
| This indicator is valid. [mean=5.08, median=6, SD=2.06] |  |  |  |  |  |  |  |  |
| This indicator is reliable. [mean=5.00, median=5, SD=1.96] |  |  |  |  |  |  |  |  |

**Patient reported unfair treatment based on ethnicity**

Proportion of patients who self-reported an experience of unfair treatment by a health professional on the basis of ethnicity

|  | 1 | 2 | 3 | 4 | 5 | 6 | 7 | N/A |
| --- | --- | --- | --- | --- | --- | --- | --- | --- |
| This indicator is important. [mean=6.31, median=7, SD=1.38] |  |  |  |  |  |  |  |  |
| This indicator is relevant to the circumpolar context. [mean=6.07, median=7, SD=1.64] |  |  |  |  |  |  |  |  |
| This indicator is valid. [mean=5.58, median=6, SD=1.73] |  |  |  |  |  |  |  |  |
| This indicator is reliable. [mean=4.75, median=5.5, SD=2.05] |  |  |  |  |  |  |  |  |

**Patient reported support during labour and birth**

Patient reported support during labour and birth (husband/partner vs. other support person)

|  | 1 | 2 | 3 | 4 | 5 | 6 | 7 | N/A |
| --- | --- | --- | --- | --- | --- | --- | --- | --- |
| This indicator is important. [mean=6.38, median=7, SD=0.96] |  |  |  |  |  |  |  |  |
| This indicator is relevant to the circumpolar context. [mean=6.29, median=7, SD=1.07] |  |  |  |  |  |  |  |  |
| This indicator is valid. [mean=5.83, median=6, SD=1.34] |  |  |  |  |  |  |  |  |
| This indicator is reliable. [mean=5.67, median=6, SD=1.44] |  |  |  |  |  |  |  |  |

**Use of analgesia in labour**

Epidural rate for vaginal deliveries

|  | 1 | 2 | 3 | 4 | 5 | 6 | 7 | N/A |
| --- | --- | --- | --- | --- | --- | --- | --- | --- |
| This indicator is important. [mean=4.81, median=5, SD=1.66] |  |  |  |  |  |  |  |  |
| This indicator is relevant to the circumpolar context. [mean=4.83, median=5, SD=1.27] |  |  |  |  |  |  |  |  |
| This indicator is valid. [mean=5.36, median=6, SD=1.29] |  |  |  |  |  |  |  |  |
| This indicator is reliable. [mean=5.64, median=6, SD=1.03] |  |  |  |  |  |  |  |  |

**Mother-infant contact at birth**

Proportion of women who reported that they were able to hold their baby within five minutes of birth (excluding infants admitted to the NICU)

|  | 1 | 2 | 3 | 4 | 5 | 6 | 7 | N/A |
| --- | --- | --- | --- | --- | --- | --- | --- | --- |
| This indicator is important. [mean=6.00, median=7, SD=1.41] |  |  |  |  |  |  |  |  |
| This indicator is relevant to the circumpolar context. [mean=5.71, median=6.50, SD=1.64] |  |  |  |  |  |  |  |  |
| This indicator is valid. [mean=5.55, median=6, SD=1.81] |  |  |  |  |  |  |  |  |
| This indicator is reliable. [mean=5.64, median=6, SD=1.29] |  |  |  |  |  |  |  |  |

**Presence of breastfeeding support programs**

Proportion of babies born in hospitals that have received the “baby friendly hospital initiative” or similar designation

|  | 1 | 2 | 3 | 4 | 5 | 6 | 7 | N/A |
| --- | --- | --- | --- | --- | --- | --- | --- | --- |
| This indicator is important. [mean=6.14, median=6, SD=0.86] |  |  |  |  |  |  |  |  |
| This indicator is relevant to the circumpolar context. [mean=6.07, median=6, SD=1.07] |  |  |  |  |  |  |  |  |
| This indicator is valid. [mean=5.17, median=5.5, SD=1.64] |  |  |  |  |  |  |  |  |
| This indicator is reliable. [mean=5.08, median=5, SD=1.62] |  |  |  |  |  |  |  |  |

**Patient reported satisfaction with care**

Proportion of women who reported being satisfied with their birth experience and care

|  | 1 | 2 | 3 | 4 | 5 | 6 | 7 | N/A |
| --- | --- | --- | --- | --- | --- | --- | --- | --- |
| This indicator is important. [mean=6.62, median=7, SD=0.51] |  |  |  |  |  |  |  |  |
| This indicator is relevant to the circumpolar context. [mean=6.62, median=7, SD=0.51] |  |  |  |  |  |  |  |  |
| This indicator is valid. [mean=6.00, median=7, SD=1.34] |  |  |  |  |  |  |  |  |
| This indicator is reliable. [mean=5.63, median=6, SD=1.57] |  |  |  |  |  |  |  |  |

**Gestational age at which patients are transferred for birth**

|  | 1 | 2 | 3 | 4 | 5 | 6 | 7 | N/A |
| --- | --- | --- | --- | --- | --- | --- | --- | --- |
| This indicator is important. |  |  |  |  |  |  |  |  |
| This indicator is relevant to the circumpolar context. |  |  |  |  |  |  |  |  |

**If you have any additional comments, please include them here.**

**ACCESSIBILITY**

The following indicators refer to healthcare accessibility. *Accessibility*refers to the ease with which health services can be reached.

**Frequency and timing of antenatal care**

Proportion of all pregnant women (with live or stillborn infants) who received antenatal care in the first trimester

|  | 1 | 2 | 3 | 4 | 5 | 6 | 7 | N/A |
| --- | --- | --- | --- | --- | --- | --- | --- | --- |
| This indicator is important. [mean=6.36, median=7, SD=1.08] |  |  |  |  |  |  |  |  |
| This indicator is relevant to the circumpolar context. [mean=6.36, median=7, SD=1.08] |  |  |  |  |  |  |  |  |
| This indicator is valid. [mean=6.25, median=6.5, SD=0.97] |  |  |  |  |  |  |  |  |
| This indicator is reliable. [mean=6.33, median=6.5, SD=0.89] |  |  |  |  |  |  |  |  |

**Prenatal care provider**

Patient reported provider for prenatal care

|  | 1 | 2 | 3 | 4 | 5 | 6 | 7 | N/A |
| --- | --- | --- | --- | --- | --- | --- | --- | --- |
| This indicator is important. [mean=6.55, median=7, SD=0.69] |  |  |  |  |  |  |  |  |
| This indicator is relevant to the circumpolar context. [mean=6.50, median=7, SD=0.67] |  |  |  |  |  |  |  |  |
| This indicator is valid. [mean=6.10, median=6, SD=0.99] |  |  |  |  |  |  |  |  |
| This indicator is reliable. [mean=5.9, median=6.0, SD=1.20] |  |  |  |  |  |  |  |  |

**Use of antenatal ultrasound**

Proportion of women who report having had at least one ultrasound in pregnancy

|  | 1 | 2 | 3 | 4 | 5 | 6 | 7 | N/A |
| --- | --- | --- | --- | --- | --- | --- | --- | --- |
| This indicator is important. [mean=6.08, median=6, SD=1.04] |  |  |  |  |  |  |  |  |
| This indicator is relevant to the circumpolar context. [mean=5.77, median=6, SD=1.54] |  |  |  |  |  |  |  |  |
| This indicator is valid. [mean=6.36, median=6, SD=0.67] |  |  |  |  |  |  |  |  |
| This indicator is reliable. [mean=6.36, median=6, SD=0.67] |  |  |  |  |  |  |  |  |

**Induced abortions**

Induced abortion ratio (CDC definition): Number of induced abortions per 1000 live births

|  | 1 | 2 | 3 | 4 | 5 | 6 | 7 | N/A |
| --- | --- | --- | --- | --- | --- | --- | --- | --- |
| This indicator is important. [mean=6.54, median=7, SD=0.52] |  |  |  |  |  |  |  |  |
| This indicator is relevant to the circumpolar context. [mean=6.46, median=6, SD=0.52] |  |  |  |  |  |  |  |  |
| This indicator is valid. [mean=6.42, median=6, SD=0.51] |  |  |  |  |  |  |  |  |
| This indicator is reliable. [mean=5.92, median=6, SD=1.08] |  |  |  |  |  |  |  |  |

**Birth attendant**

Proportion of women giving birth with a skilled birth attendant

|  | 1 | 2 | 3 | 4 | 5 | 6 | 7 | N/A |
| --- | --- | --- | --- | --- | --- | --- | --- | --- |
| This indicator is important. [mean=6.43, median=7, SD=0.93] |  |  |  |  |  |  |  |  |
| This indicator is relevant to the circumpolar context. [mean=5.86, median=6, SD=1.23] |  |  |  |  |  |  |  |  |
| This indicator is valid. [mean=6.17, median=6, SD=0.94] |  |  |  |  |  |  |  |  |
| This indicator is reliable. [mean=6.17, median=6, SD=0.94] |  |  |  |  |  |  |  |  |

**FHR monitoring during labour**

Patient reported use of electronic fetal monitoring (continuous vs. intermittent vs. none)

|  | 1 | 2 | 3 | 4 | 5 | 6 | 7 | N/A |
| --- | --- | --- | --- | --- | --- | --- | --- | --- |
| This indicator is important. [mean=5.23, median=5, SD=0.93] |  |  |  |  |  |  |  |  |
| This indicator is relevant to the circumpolar context. [mean=5.31, median=5, SD=0.95] |  |  |  |  |  |  |  |  |
| This indicator is valid. [mean=5.70, median=6, SD=0.67] |  |  |  |  |  |  |  |  |
| This indicator is reliable. [mean=5.70, median=6, SD=1.16] |  |  |  |  |  |  |  |  |

**Place or setting for birth**

Distribution of births by location and size of maternity unit (home vs. maternity unit stratified by number of births/year)

|  | 1 | 2 | 3 | 4 | 5 | 6 | 7 | N/A |
| --- | --- | --- | --- | --- | --- | --- | --- | --- |
| This indicator is important. [mean=6.08, median=6, SD=1.38] |  |  |  |  |  |  |  |  |
| This indicator is relevant to the circumpolar context. [mean=6.23, median=7, SD=1.17] |  |  |  |  |  |  |  |  |
| This indicator is valid. [mean=6.08, median=6, SD=.090] |  |  |  |  |  |  |  |  |
| This indicator is reliable. [mean=5.92, median=6, SD=1.00] |  |  |  |  |  |  |  |  |

**Travel to place of birth**

Proportion of women that report having to travel >100km to place of birth

|  | 1 | 2 | 3 | 4 | 5 | 6 | 7 | N/A |
| --- | --- | --- | --- | --- | --- | --- | --- | --- |
| This indicator is important. [mean=6.71, median=7, SD=0.47] |  |  |  |  |  |  |  |  |
| This indicator is relevant to the circumpolar context. [mean=6.87, median=7, SD=0.38] |  |  |  |  |  |  |  |  |
| This indicator is valid. [mean=6.33, median=6, SD=0.65] |  |  |  |  |  |  |  |  |
| This indicator is reliable. [mean=6.25, median=6, SD=0.87] |  |  |  |  |  |  |  |  |

**Postpartum visit(s)**

Proportion of women that report having attended a postpartum follow up visit

|  | 1 | 2 | 3 | 4 | 5 | 6 | 7 | N/A |
| --- | --- | --- | --- | --- | --- | --- | --- | --- |
| This indicator is important. [mean=6.36, median=6, SD=0.63] |  |  |  |  |  |  |  |  |
| This indicator is relevant to the circumpolar context. [mean=6.08, median=6, SD=1.38] |  |  |  |  |  |  |  |  |
| This indicator is valid. [mean=5.91, median=6, SD=1.30] |  |  |  |  |  |  |  |  |
| This indicator is reliable. [mean=6.09, median=6, SD=0.94] |  |  |  |  |  |  |  |  |

**Availability of a maternity care provider in patient's community**

|  | 1 | 2 | 3 | 4 | 5 | 6 | 7 | N/A |
| --- | --- | --- | --- | --- | --- | --- | --- | --- |
| This indicator is important. |  |  |  |  |  |  |  |  |
| This indicator is relevant to the circumpolar context. |  |  |  |  |  |  |  |  |

**Availability of a maternity care provider that speaks the same language and/or is from the same culture as the patient**

|  | 1 | 2 | 3 | 4 | 5 | 6 | 7 | N/A |
| --- | --- | --- | --- | --- | --- | --- | --- | --- |
| This indicator is important. |  |  |  |  |  |  |  |  |
| This indicator is relevant to the circumpolar context. |  |  |  |  |  |  |  |  |

**If you have any additional comments, please include them here.**

**COST**

The following indicators refer to healthcare cost/expenditure. Many important ideas were raised regarding the cost of maternity care in circumpolar regions. Some of these ideas were incorporated into a single example indicator (below). Other possible cost indicators could be developed from the ideas you raised and should perhaps be the subject of future work.

**Cost of maternity care per patient**

Unit cost of maternity (adjusted for case mix and market forces)

|  | 1 | 2 | 3 | 4 | 5 | 6 | 7 | N/A |
| --- | --- | --- | --- | --- | --- | --- | --- | --- |
| This indicator is important. [mean=5.57, median=6, SD=1.34] |  |  |  |  |  |  |  |  |
| This indicator is relevant to the circumpolar context. [mean=5.85, median=6, SD=1.28] |  |  |  |  |  |  |  |  |
| This indicator is valid. [mean=4.92, median=6, SD=1.85] |  |  |  |  |  |  |  |  |
| This indicator is reliable. [mean=4.62, median=5, SD=1.89] |  |  |  |  |  |  |  |  |

**Maternal Length of stay**

Proportion of women staying < 3 days in hospital after childbirth

|  | 1 | 2 | 3 | 4 | 5 | 6 | 7 | N/A |
| --- | --- | --- | --- | --- | --- | --- | --- | --- |
| This indicator is important. [mean=5.71, median=6, SD=0.91] |  |  |  |  |  |  |  |  |
| This indicator is relevant to the circumpolar context. [mean=5.53, median=6, SD=1.20] |  |  |  |  |  |  |  |  |
| This indicator is valid. [mean=5.62, median=6, SD=1.39] |  |  |  |  |  |  |  |  |
| This indicator is reliable. [mean=5.62, median=6, SD=1.39] |  |  |  |  |  |  |  |  |

**Neonatal length of stay**

Proportion of neonates being discharged from hospital within 48 hours of birth

|  | 1 | 2 | 3 | 4 | 5 | 6 | 7 | N/A |
| --- | --- | --- | --- | --- | --- | --- | --- | --- |
| This indicator is important. [mean=5.57, median=6, SD=0.94] |  |  |  |  |  |  |  |  |
| This indicator is relevant to the circumpolar context. [mean=5.46, median=6, SD=1.20] |  |  |  |  |  |  |  |  |
| This indicator is valid. [mean=5.31, median=6, SD=1.38] |  |  |  |  |  |  |  |  |
| This indicator is reliable. [mean=5.46, median=6, SD=1.45] |  |  |  |  |  |  |  |  |

**Total cost to obtain standard of care for (example condition) including cost of medical evacuation as well as direct and indirect costs incurred by the patient/family**

|  | 1 | 2 | 3 | 4 | 5 | 6 | 7 | N/A |
| --- | --- | --- | --- | --- | --- | --- | --- | --- |
| This indicator is important. |  |  |  |  |  |  |  |  |
| This indicator is relevant to the circumpolar context. |  |  |  |  |  |  |  |  |

**If you have any additional comments, please include them here.**
